# *Prognathodes basabei*, a new species of butterflyfish (Perciformes, Chaetodontidae) from the Hawaiian Archipelago

**DOI:** 10.3897/zookeys.614.10200

**Published:** 2016-09-06

**Authors:** Richard L. Pyle, Randall K. Kosaki

**Affiliations:** 1 Bernice P. Bishop Museum, 1525 Bernice Street, Honolulu, Hawai‘i 96817, USA; 2 NOAA Papahānaumokuākea Marine National Monument, 1845 Wasp Blvd, Building 176, Honolulu, Hawai‘i 96818, USA

**Keywords:** Mesophotic Coral Ecosystem, Closed-Circuit Rebreather, Endemic, Papahānaumokuākea Marine National Monument

## Abstract

A new species of the butterflyfish genus *Prognathodes* is described from specimens collected at a depth of 55–61 m off Pearl and Hermes Atoll, Northwestern Hawaiian Islands. This species has been observed by mixed-gas divers and from submersibles at depths ranging from 45–187 m throughout the Hawaiian Archipelago, with shallower sightings in the Northwestern Hawaiian Islands and deeper in the Main Hawaiian Islands. It is similar to *Prognathodes guezei* (Maugé and Bauchot 1976) from the western Indian Ocean, and at least one other undescribed species of *Prognathodes* from Palau, differing from these species in the number of soft dorsal-fin rays, size of head, and body depth. There are also differences in the life color, and a substantial genetic difference from the Palauan species (d » .08 in mtDNA cytochrome oxidase I).

## Introduction

The genus *Prognathodes* Gill, 1862 (type species *Chelmo pelta* Günther, 1860 = *Chaetodon aculeatus* Poey, 1860) currently includes eleven valid species: seven from the Atlantic, two from the tropical eastern Pacific, and two from the Indian Ocean and western Pacific. Pyle and Chave (1994) first reported the presence of a species of this genus in the Hawaiian Islands at depths of 106–187 m, based on video and observations from research submersibles operated by the Hawaii Undersea Research Laboratory (HURL). They noted its similarity to *Prognathodes guezei* (Mauge and Bauchot 1976), a species then known only from the two type specimens collected at a depth of 80 m off Réunion Island in the western Indian Ocean.

While conducting an exploratory dive using a mixed-gas closed-circuit rebreather off the south shore of O‘ahu (Main Hawaiian Islands) on 17 May 1998, the senior author (RLP) observed (but was unable to collect) a group of three *Prognathodes* near an undercut limestone ledge at a depth of 114 m. Two weeks later (30 May 1998), with the assistance of Peter K. Basabe, RLP collected the first specimen of this species at a depth of 120 m near Kealakekua Bay on the Kona coast of the island of Hawai‘i (TenBruggencate 1998). The following day he collected several more individuals at a depth of 115 m near the site of the observation of 17 May (Allen et al. 1998). All of the collected individuals were brought to the surface alive and maintained in captivity. Unfortunately, when they eventually died, only one was preserved, and it was too badly deteriorated to serve as a type specimen.

In the years that followed, several more individuals of this species were collected from depths of 115–125 m around O‘ahu by mixed-gas rebreather divers. All were maintained in aquaria until their deaths, but none were properly preserved as suitable type specimens. On 27 April 2007, RLP collected two individuals of a similar species of *Prognathodes* at a depth of 116 m at Palau (Republic of Belau), in the western Pacific. Although some color differences between the Palauan and Hawaiian fishes were noted, the authors felt it was necessary to obtain specimens of the Hawaiian population for comparison of morphological and genetic characters before determining whether they are the same species.

In August of 2009, after the establishment of the Papahānaumokuākea Marine National Monument in the Northwestern Hawaiian Islands, the U.S. National Oceanic and Atmospheric Administration (NOAA) began a series of annual surveys of Mesophotic Coral Ecosystems (MCEs) within the Monument using mixed-gas diving technology. During the first of these surveys, the authors collected a group of three individuals of the unidentified *Prognathodes* at a depth of 61 m off the SW side of Pearl and Hermes Atoll. Unfortunately, tissue samples taken from these specimens were misplaced, so it was not possible to make genetic comparisons with the Palauan population.

Finally, in September of 2015, the authors were able to collect three more specimens at the same site off Pearl and Hermes Atoll where the three specimens had been collected in 2009, and obtain additional tissue samples for genetic analyses. Based on an examination of both morphological and genetic characters of the six Hawaiian specimens, as well as comparisons with the two specimens from Palau and *Prognathodes guezei*, we can now confirm that the Hawaiian population represents a new species, distinct from both *Prognathodes guezei* and the undescribed Palauan species. We herein describe the Hawaiian population as the new species, *Prognathodes basabei*.

## Methods

Specimens were collected with hand nets during deep dives using mixed-gas, closed-circuit rebreathers. Additional observations, videos and images of this species were made from the two *Pisces* submersibles operated by the Hawaii Undersea Research Laboratory (HURL, at the University of Hawai‘i), and by mixed-gas rebreather divers in the Main Hawaiian Islands and Northwestern Hawaiian Islands.

Standard length (SL) was measured from the tip of the snout to the caudal-fin base. Total length (TL) was measured from the tip of the snout to the posterior edge of the caudal fin. Head length was measured from the tip of the snout to the posterior-most edge of the fleshy flap near the upper end of the gill opening. Body depth is the greatest depth of body measured as a vertical from the ventral edge of the abdomen to the upper edge of scaled fleshy sheath of the dorsal fin (typically from about fourth or fifth dorsal spine). Width of the body is the maximum width. Snout length is the distance from the tip of the snout to the closest point on the bony orbit. Predorsal length is the distance from the tip of the snout to the angle formed by the scaled fleshy sheath at the insertion point of the first dorsal-fin spine, when erected. Preanal length is the distance from the tip of the snout to angle formed by the scaled fleshy sheath at the insertion point of the first anal-fin spine, when erected. The base of the dorsal fin is measured from the extreme base of the first dorsal-fin spine to the extreme base of the last dorsal-fin soft ray. The base of the anal fin is measured from the extreme base of the first anal-fin spine to the extreme base of the last anal-fin soft ray. Orbit diameter is the maximum diameter of the bony orbit. Interorbital width is the width of the bony interorbital space. Depth of the caudal peduncle is the least depth. Pelvic-fin spine length was measured from the extreme base of the pelvic-fin spine to its distal tip. Pelvic fin length was only measured on specimens with intact filamentous extensions of the first pelvic-fin soft ray, and represents the length of that ray from its extreme base to the distal tip of the filamentous extension. Length of spines and soft rays of dorsal and anal fins were measured from the extreme base to the most distal tip. Caudal fin length was defined as the difference between TL and SL. Pectoral fin length was measured as the longest fin ray, from its extreme base to its distal tip.

The last dorsal- and anal-fin soft rays are branched to the base and were counted as a single ray. Caudal-fin ray counts include small unsegmented and rudimentary rays. Pectoral-fin ray counts include first and last unsegmented rays. Pored lateral-line scale counts include only those scales with pores. Scale-row counts above and below lateral line to origins of dorsal and anal fins (respectively) include small truncate scales at bases of respective fins. Vertebral counts include the first vertebra fused to the skull, and the last vertebra fused to the hypural plate.

All counts and measurements except vertebrae were made directly from specimens. Measurements were made using dial calipers with +/- 0.05 mm precision. Lengths of dorsal- and anal-fin spines and soft rays were made with the aid of a bright light transmitted from behind the fins to reveal the position of their extreme bases. Vertebral counts were made from x-radiographs.

Head length, depth of body, width of body, snout length, predorsal length, preanal length, length of dorsal-fin and anal-fin bases, orbit diameter, interorbital width, caudal peduncle depth, and lengths of fin spines and rays are expressed as percent of SL. Counts and measurements for paratypes, if different from the holotype, are presented in parentheses after the value for the holotype.

The holotype and three paratypes have been deposited at the Bernice Pauahi Bishop Museum fish collection, Honolulu (BPBM), and paratypes have been deposited at the California Academy of Sciences fish collection, San Francisco (CAS), and the U.S. National Museum of Natural History, Washington, D.C. (USNM).

Tissue samples were obtained from the holotype and two paratypes (CAS 242132 and USNM 440272). DNA barcodes (cytochrome c oxidase I; COI) were sequenced following the protocol described in Copus et al. (2015). GenBank accession numbers and Barcode of Life Database (BOLD) identifiers for DNA sequences are presented along with museum catalog numbers for type material and non-type specimens.

## Taxonomy

### Prognathodes basabei

Taxon classificationAnimaliaPerciformesChaetodontidae

Pyle & Kosaki sp. n.

http://zoobank.org/A843AA98-2312-4E9E-B1EC-E28B5478085E

**Figure 1.**
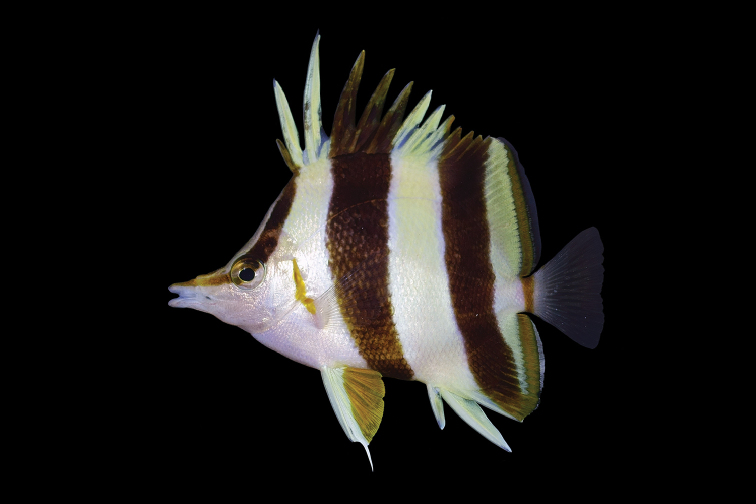
Holotype of *Prognathodes basabei* (BPBM 41290), collected at a depth of 61 m off Pearl and Hermes Atoll, Northwestern Hawaiian Islands. Photo by R. L. Pyle.

**Figure 2.**
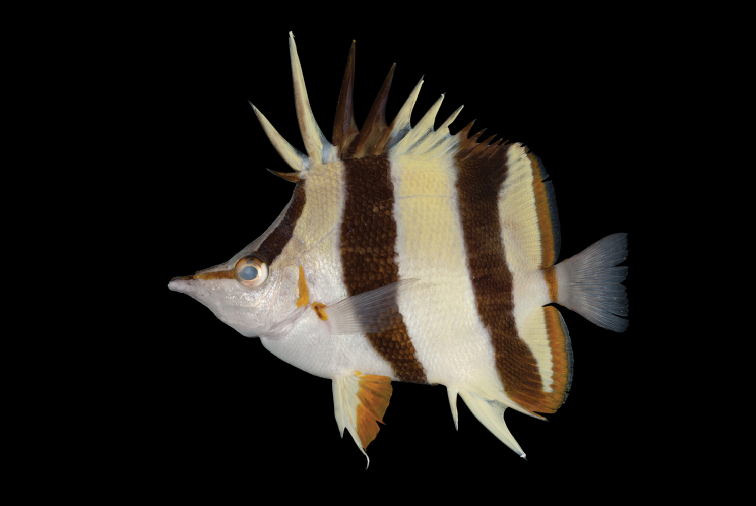
Paratype of *Prognathodes basabei* (BPBM 41285-1), collected at a depth of 55 m off Pearl and Hermes Atoll, Northwestern Hawaiian Islands. Photo by R. L. Pyle.

**Figure 3.**
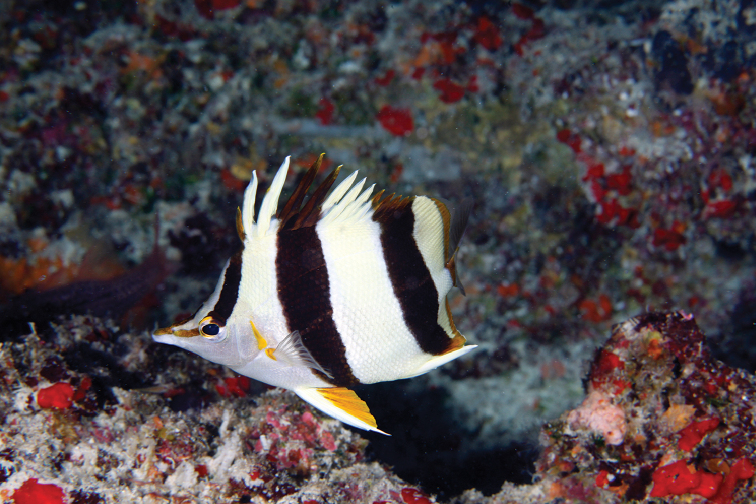
*Prognathodes basabei* at a depth of approximately 55 m off Pearl and Hermes Atoll, Northwestern Hawaiian Islands. Photo by G. McFall.

**Figure 4.**
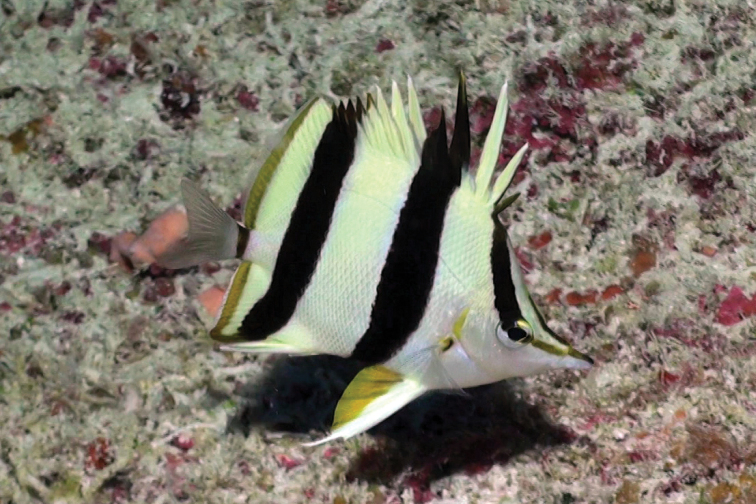
*Prognathodes basabei* at a depth of 90 m off Pearl and Hermes Atoll, Northwestern Hawaiian Islands. Photo by R. L. Pyle.

**Figure 5.**
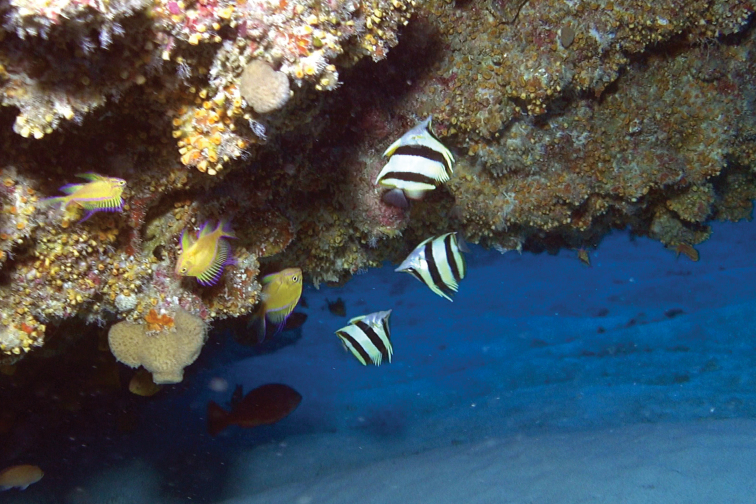
A group of three *Prognathodes basabei* at a depth of 90 m off Pearl and Hermes Atoll, Northwestern Hawaiian Islands. Photo by R. L. Pyle.

Figs 1 , 2 , 3 , 4 , 5

Prognathodes sp. 1; Allen et al. 1998: 250.
Prognathodes “basabei” ; Randall 2007: 291.

#### Type locality.

Northwestern Hawaiian Islands, Pearl and Hermes Atoll, southwest side, “Prognathodes Point”, 27.7641°N, 175.9859°W.

#### Holotype.

BPBM 41290, female, GenBank KX783257, Barcode of Life PROBA001-16, 93.4 mm SL, Northwestern Hawaiian Islands, Pearl and Hermes Atoll, southwest side, “Prognathodes Point”, 27.7641°N, 175.9859°W, 61 m, 13 September 2015, R. L. Pyle, aboard NOAA ship *Hi‘ialakai* (Cruise: HA-15-05), hand nets, under limestone ledge (ancient seashore). Collected as part of a group of three associated individuals (along with CAS 242132 and USNM 440272).

#### Paratypes.

BPBM 41285, 3 specimens: 97.7–106.3 mm SL, same location, habitat, collector, vessel and collecting method as holotype, 55 m, 17 August 2009, Cruise: HI-09-06; CAS 242132, GenBank KX783255, Barcode of Life PROBA003-16, 102.5 mm SL, same location, depth, habitat, collector, vessel, cruise and collecting method as holotype, 14 September 2015; USNM 440272, GenBank KX783256, Barcode of Life PROBA002-16, same data as holotype.

#### Non-type specimen.

BPBM 38441, 82 mm SL, Hawaiian Islands, O‘ahu, south shore, 116 m, 31 May 1998, R. L. Pyle, hand nets, along limestone ledge (specimen died in captivity and partially deteriorated).

#### Diagnosis.

A species of *Prognathodes* (*sensu* Smith et al. 2003) distinguished by the following combination of characters: dorsal-fin soft rays 21 or 22; anal-fin soft rays 16 or 17; head 2.63–2.80 in SL; body depth 1.58–1.69 in SL; pelvic-fin spine length 3.63–4.07 in SL; color in life pale yellow dorsally fading to white ventrally (sometimes entirely white) with three black bands with narrow white margins on each side of the body, the first band originating at and including the first dorsal-fin spine, extending diagonally to the eye and continuing horizontally as an orangish brown stripe from the eye to the tip of the snout, the second band originating at and including the fourth to sixth dorsal-fin spines, extending vertically at a slightly posterior angle to the ventral surface of the abdomen just anterior to the anus, tapering slightly and curving slightly posteriorly below the pectoral fin, and the third band originating at and including the last four to five dorsal-fin spines and first four to five dorsal-fin soft rays, extending vertically at a slightly posterior angle to and including the first several anal-fin soft rays, a narrow orange band on the dorso-posterior margin of the operculum, extending ventrally the posterior angle of the operculum, an oblong orange spot with some dark pigmentation on the upper one-third of the pectoral-fin axis, pelvic fins white on the spine and anterior one-third of fin, and bright orange on the posterior two-thirds of fin, a bright orange submarginal band with narrow white posterior margin extending along the posterior margins of the soft portions of the dorsal and anal fins, and continuing across the caudal peduncle.

#### Description.

Dorsal fin XIII (XII in two paratypes),21 (22 in one paratype), last soft ray branched to base; anal fin III,16 (17 in one paratype), last soft ray branched to base; pectoral-fin rays 16 (15 in one paratype); pelvic-fin rays I,5; principal branched caudal rays 15, upper procurrent unbranched caudal rays 4, lower procurrent unbranched caudal rays 3; pored lateral-line scales 26 (24–28); scale rows above lateral line to origin of dorsal fin 10 (11 in all but one paratype); scale rows below lateral line to origin of anal fin 24 (21–27); gill rakers on upper limb 6, on lower limb 9 (10 in one paratype); vertebrae 24.

Body deep, the depth 1.58 (1.61–1.69) in SL, and compressed, the width 4.05 (3.80–4.33) in depth; head length 2.63 (2.65–2.80) in SL; snout produced, its length 2.35 (2.19–2.62) in head; orbit diameter 3.59 (3.50–3.83) in head; interorbital slightly convex, the least bony width 4.18 (3.85–4.28) in head; least depth of caudal peduncle 4.33 (4.00–4.33) in head.

Mouth small, the upper jaw 2.77 in head, slightly diagonal, the gape forming an angle of about 20° to the horizontal, the upper jaw slightly protruding; teeth in jaws densely setiform, the longest 7.8 in orbit diameter; nostrils anterior to the eye horizontally in line with the top of the iris, the anterior in a short membranous tube with a well-developed posterior flap, the posterior slightly larger, ovate, with a low fleshy rim. Lower edge of lacrimal smooth; margin of preopercle finely serrate; margins of other opercular bones smooth.

Lateral line forming a broad arc, ending below the base of the third to fifth soft dorsal ray and within the second black band on the body. Scales ctenoid, moderately large on body except for chest and near origins of dorsal and anal fins, where small; head fully scaled except anterior portions of both jaws and around nostrils, the scales on the head small; scales on fleshy sheath surrounding base of dorsal and anal fins moderately large anteriorly and proximally, reducing in size posteriorly and distally; scales on caudal peduncle and covering base of caudal fin small.

Origin of dorsal fin slightly posterior to upper end of gill opening, its base 1.45 (1.43–1.52) in SL; first dorsal-fin spine the shortest, its length 3.09 (2.49–3.68) in head; second dorsal-fin spine 1.27 (1.42–1.98, broken in one paratype) in head; third dorsal-fin spine the longest, its length 0.93 (0.94–1.11, broken in one paratype) in head; fourth dorsal-fin spine nearly as long as the third, its length 1.04 (1.03–1.13, broken in one paratype, deformed in one paratype) in head; fifth dorsal-fin spine shorter, its length 1.20 (1.14–1.23, broken in one paratype) in head; dorsal-fin spines progressively shorter posteriorly, the last 1.80 (1.80–2.06) in head; membranes between anterior dorsal-fin spines deeply incised, progressively less so posteriorly; first dorsal-fin soft ray the longest, approximately the same length as the last dorsal-fin spine, 1.80 (1.70–1.88) in head, dorsal-fin soft rays progressively shorter posteriorly; first anal-fin spine the shortest, its length 2.54 (2.44–3.10) in head; second anal-fin spine the longest, its length 1.31 (1.16–1.31) in head; third anal-fin spine 1.52 (1.39–1.62) in head; first anal-fin soft ray the longest, its length 1.35 (1.39–1.62) in head, anal-fin soft rays progressively shorter posteriorly; caudal fin slightly convex with a slight concavity at the mid-line, its length 1.79 (1.83–2.00) in head; pectoral fins 1.37 (1.26–1.43) in head; pelvic spine 1.38 (1.36–1.51) in head; first soft ray of pelvic fin with a filamentous extension, its length 1.08 (1.19, broken in all but one paratype) in head.

Color in life as in Figures 1–5. Body pale yellow dorsally fading to white ventrally and on the thorax and lower head (color of body sometimes lacking pale yellow coloration); three prominent black bands on each side of the body with narrow white margins, the first band originating at and including the first dorsal-fin spine, extending diagonally to the eye and continuing horizontally as an orangish brown stripe from the eye to the tip of the snout, the second band originating at and including the fourth to sixth dorsal-fin spines, extending vertically at a slightly posterior angle to the ventral surface of the abdomen just anterior to the anus, tapering slightly and curving slightly posteriorly below the pectoral fin, and the third band originating at and including last four to five dorsal-fin spines and first four to five soft dorsal rays, extending vertically at a slightly posterior angle to and including the first several anal soft rays, the bands becoming dark orangish brown distally on the dorsal fin; a narrow orangish brown stripe extending from the dorsal side of the snout broadening dorsally on the nape to a point just above the interorbital space, becoming darker dorsally; a narrow orange band on the dorso-posterior margin of the operculum, extending ventrally to the posterior angle of the operculum, an oblong orange spot with some dark pigmentation on the upper one-third of the pectoral-fin axis; pelvic fins white on the spine and anterior one-third of fin, and bright orange on the posterior two-thirds of fin; a bright orange submarginal band with narrow white posterior margin extending along the posterior margins of the soft portions of the dorsal and anal fins and continuing across the caudal peduncle; caudal fin and pectoral fins translucent.

**Figure 6.**
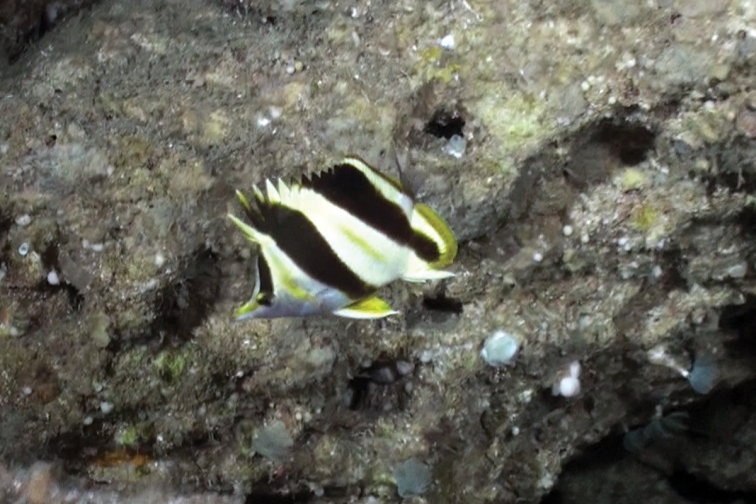
*Prognathodes guezei* at a depth of 117 m off Sodwana Bay, South Africa. Photo by R. L. Pyle.

Color in alcohol similar to life color, except body a uniform dull yellow, bands dark brown, and orange areas pale brown.

A single juvenile, about 25 mm SL, was observed by RLP at a depth of 120 m during a dive off Pearl Harbor, O‘ahu on 16 August 1998. The general body shape and color pattern were the same as for adults.

Morphometric data for selected characters of type specimens are provided in Table 1.

**Table 1.** Morphometric and meristic data for selected characters of type specimens of *Prognathodes basabei*. Values of morphometric data (other than TL and SL) are represented as % of SL.

	Holotype	Paratypes				
Morphometrics	BPBM 41290	BPBM 41285-1	BPBM 41285-2	BPBM 41285-3	CAS 242132	USNM 440272
Sex	Female	Female	Male	Male	Male	Male
Total length (TL) in mm	113.2	125.5	123.1	117.8	122.5	119.6
Standard length (SL) in mm	93.4	106.3	102.2	97.7	102.5	99.8
Head length	38	36	38	38	36	38
Body depth	63	59	60	62	62	62
Body width	16	14	16	14	16	16
Snout length	16	16	17	17	15	14
Predorsal length	48	43	44	42	45	46
Preanal length	75	75	77	73	76	77
Base of dorsal fin	69	66	66	68	68	70
Base of anal fin	33	32	31	33	32	30
Orbit diameter	11	9.8	11	9.8	9.9	11
Interorbital Width	9.1	9.1	8.8	9.0	9	10
Caudal Peduncle Depth	8.8	9.0	8.7	8.9	8.5	8.7
Pelvic Spine	28	25	25	26	26.3	25
Pelvic Fin	35	-	-	32	-	-
First Dorsal Spine length	12	12	13	10	14	11
Second Dorsal Spine length	30	25	broken	27	18	23
Third Dorsal Spine length	41	38	broken	39	38	34
Fourth Dorsal Spine length	37	35	damaged	37	broken	33
Fifth Dorsal Spine length	32	32	31	31	broken	31
Last Dorsal Spine length	21	19	21	21	17	18
Longest Dorsal Ray length	21	21	22	21	21	20
First Anal Spine length	15	13	12	13	15	13
Second Anal Spine length	29	29	30	29	31	29
Third Anal Spine length	25	26	23	26	24	26
Longest anal ray length	28	26	24	26	23	23
Caudal fin length	21	18	20	21	20	20
Pectoral fin length	28	25	27	27	28	27
**Meristics**						
Dorsal Spines	XIII	XIII	XII	XII	XIII	XIII
Dorsal rays	21	21	22	21	21	21
Anal Spines	3	3	3	3	3	3
Anal Rays	16	16	17	16	16	16
Pectoral Rays	16	15	16	16	16	16
Caudal Rays	22	22	22	22	22	22
Pored lateral line scales	26	28	25	24	24	27
Dorsal scale rows	10	10	11	11	11	11
Ventral scale rows	24	27	21	23	24	25
Gill rakers	6+9	6+9	6+9	6+10	13	13

#### Distribution.

*Prognathodes basabei* has been observed or collected at depths of 45–187 m at several locations throughout the Hawaiian Archipelago, including both the main Hawaiian Islands (Hawai‘I, O‘ahu, Penguin Banks) and the Northwestern Hawaiian Islands (NWHI; French Frigate Shoals, Lisianski, Pearl and Hermes Atoll, Midway Atoll, Kure Atoll). No observations of this species were made during 61 submersible dives or eight mixed-gas rebreather dives to appropriate depths at Johnston Atoll (Randall et al. 1985; Wagner et al. 2014), nor has any similar fish been observed or collected anywhere in the central or eastern Pacific. Thus, it appears that *Prognathodes basabei* is endemic to the Hawaiian Archipelago (although further exploration of MCEs in nearby regions may yet reveal its presence elsewhere). This is consistent with the observation that fish assemblages on deep coral reefs have proportionally more endemic species than on shallow reefs (Pyle 1996, Kane et al. 2014, Kosaki et al. 2016).

#### Habitat.

Pyle and Chave (1994: 92) described the habitat for this species based on videotaped observations from submersibles as follows:

*Eighteen (56%) of the observed [fish] were in areas of basalt substrata (e.g., basalt talus, blocky lava, lava tubes and pillows, basalt boulders), 13 (41 %) were in limestone habitats (primarily limestone holes and ledges), and one fish was sighted on a large (2-m diam.) water pipe. Four of the fish were in the vicinity of an unidentified antipatharian coral, three near* Cirrhipathes spiralis *(Linnaeus), and one near* Antipathes dichotoma *Pallas*.

Subsequent observations of this species by divers and submersible dives, totaling several dozen individuals mostly off O‘ahu and various sites within the NWHI, were all found in association with limestone ledges and discontinuities representing ancient shorelines (Figures 3–5). In almost all cases, the fish were found underneath, inside of, or in close proximity to small undercut overhangs or caves, often swimming upside-down in association with the roof of the overhangs and caves. There are no obvious associations with other species, such as antipathinarian corals, other corals and sessile invertebrates, or particular fish species; although certain other fish species, such as than anthias *Odontanthias fuscipinnis* (Jenkins, 1901) and the wrasse *Bodianus sanguineus* (Jordan & Evermann, 1903), tend to occupy the same depth and habitat.

#### Etymology.

We take great pleasure in naming this species *basabei*, in honor of Peter K. Basabe, long-time diver, aquarium fish collector and resident of Kona, Hawai‘i, both for his role in the collection of the first specimen of this new species in 1998, and more generally for his extensive contributions and assistance to many researchers (especially the authors) in the ichthyological community.

#### Morphological comparisons.

*Prognathodes basabei* appears to be most similar in color and morphology to an undescribed *Prognathodes* species collected at a similar depth in Palau. These two species differ from each other in number of dorsal-fin soft rays (21–22 for *basabei*, compared to 17–19 for the Palau species) and anal-fin soft rays (16–17, compared to 15). *Prognathodes basabei* also has a smaller head (2.63–2.80 in SL, compared to 2.48–2.49 in SL), deeper body (1.58–1.69 in SL, compared to 1.71–1.76 in SL), and shorter pelvic-fin spine (3.63–4.07 in SL, compared to 4.18–4.46 in SL) than the Palau species. The two species also differ in certain aspects of life color. The anterior edge of the second black band of the Palau species originates at the third dorsal-fin spine, whereas this band originates on the fourth dorsal-fin spine in *Prognathodes basabei*. Moreover, both of the dark bands on the Palau species are proportionally broader dorsally, tapering more substantially ventrally than in *Prognathodes basabei*. Also, the orangish coloration on the pelvic fins and posterior margin of the soft dorsal and anal fins of the Palau species are much darker and brownish than in *Prognathodes basabei*.

*Prognathodes basabei* is also similar in color and morphology to *Prognathodes guezei* from the western Indian Ocean. It differs from that species morphologically in number of dorsal-fin soft rays (21–22 for *basabei*, compared to 20 for *guezei*), head size (2.63–2.80 in SL, compared to 2.47–2.48 in SL), body depth (1.58–1.69 in SL, compared to 1.87–1.95 in SL), and shorter pelvic-fin spine (3.63–4.07 in SL, compared to 4.21–4.33 in SL). There are also several differences in life color between the two species. In particular, *Prognathodes guezei* (Figure 6) has more pronounced and discrete yellow bars on the body between the black bands, compared with more diffuse and paler yellow in *Prognathodes basabei*. As with the Palau species, the anterior edge of the second black band of the *Prognathodes guezei* originates at the third dorsal-fin spine, whereas this band originates on the fourth dorsal-fin spine in *Prognathodes basabei*, and the two black bands on the body of *Prognathodes guezei* taper even more substantially than they do in the Palau species, with the dorsal end of the posterior band in *Prognathodes guezei* covering the last five dorsal-fin spines, compared with the last four dorsal-fin spines on *Prognathodes basabei*. Also, the orangish coloration on the pelvic fins and posterior margin of the soft dorsal and anal fins of *Prognathodes guezei* are much paler and yellowish than in *Prognathodes basabei*.

#### Genetic comparisons.

Genetic comparisons provide another compelling justification for regarding *Prognathodes basabei* as distinct from the Palau species. The vertebrate mtDNA barcode (cytochrome oxidase I) sequences obtained from the holotype and two paratypes of *Prognathodes basabei*, compared to specimens of *Prognathodes* sp. collected in Palau, reveal 8% uncorrected sequence divergence. This is consistent with species-level divergences in other fish taxa (Johns and Avise 1998, Bellwood et al. 2004, Fessler and Westneat 2007, Randall and Rocha 2009, Rocha 2004, Rocha et al. 2008). The accepted mtDNA clock rate of approximately 2% per million years in fishes (Bowen et al. 2001, Reece et al. 2010) indicates divergence between these species on the order of 4 million years.

No tissue samples or DNA sequences have been reported for *Prognathodes guezei*, but given the geographic distributions of *Prognathodes guezei* in the western Indian Ocean, the Palau species, and *Prognathodes basabei*, we anticipate that the genetic divergence between *Prognathodes basabei* and *Prognathodes guezei* will prove to be even deeper than that between *Prognathodes basabei* and the Palau species.

#### Discussion.

*Prognathodes basabei* is an example of the conspicuous new fish species that have been discovered on deep coral reefs over the past two decades, mostly involving the use of modern mixed-gas closed-circuit rebreather diving technology (Pyle 1996, 2000). There has been increased attention focused on mesophotic coral ecosystems (MCEs), coral-reef habitat at depths of approximately 30–150 m in tropical regions worldwide (Hinderstein et al. 2010, Baker et al. 2016).

One particularly unusual characteristic of this species is the tendency for it to be found in groups of three individuals. Although Pyle and Chave (1994) reported that most videotaped observations from submersibles involved apparent pairs or solitary individuals, in most cases these observations were incidental to the research focus on the submersible dives, so no concerted effort was made to determine the total number of individuals at each sighting. Every observation of adults of this species by the authors during mixed-gas dives in both the main Hawaiian Islands and NWHI (nearly two dozen instances), as well as observations by RLP during several submersible dives off south O‘ahu in 2011, involved groups of three individuals; the only exception was the solitary juvenile observed by RLP in 1998. Only two individuals of the Palau species were observed together, and none of the approximately ten individuals of *Prognathodes guezei* observed by RLP at depths of 115–120 m off Sodwana Bay in 2011 were found in a group of three. Butterflyfishes (Chaetodontidae) in general are known to display a variety of social and mating systems, including monogamous pairs, harems, and schools (Reese 1975, Yabuta and Berumen 2013). Territoriality and the distribution of food resources are important determinants of these social systems (Hourigan 1988, Pratchett et al. 2013). Groups of three individuals as a primary social grouping have not been noted in other chaetodontid species. The six type specimens of *Prognathodes basabei* were found as two groups of three individuals. In both cases, the groupings included a single female and two males. More samples are necessary to determine whether such associations represent loose social groupings, territorial behavior, a mating system, or coincidence.

Another interesting aspect of this new species is the strikingly similar color pattern it shares with both the Palau species, and with *Prognathodes guezei*, in contrast to the deep genetic divergence that exists between the Hawaiian and Palauan specimens. It will be interesting to compare the genetics of *Prognathodes guezei* once tissue samples can be obtained, and as previously noted, we expect the genetic divergence to be similarly deep. A more thorough analysis and discussion of genetic comparisons between *Prognathodes basabei* and the Palauan species will be included in the forthcoming description of the latter species.

*Prognathodes basabei* is the twelfth recognized member of the genus, a group generally inhabiting deeper habitats than most other chaetodontid species. Nalbant (1995) suggested that the group may have an antitropical distribution, which would apply to *Prognathodes basabei* in Hawai‘i and *Prognathodes guezei* in the southwest Indian Ocean, but less so in light of the undescribed species in Palau. One potential explanation for such disjunct distributions is that these are relics of a once more widely distributed genus (paleoendemics; Bellwood and Meyer 2009). Another, perhaps more likely explanation is that the dearth of mesophotic exploration across the tropical central and western Pacific and Indian Oceans has left significant gaps in our understanding of *Prognathodes* distribution, and that additional populations and species await discovery.

## Supplementary Material

XML Treatment for Prognathodes basabei
